# PURPL represses autophagic cell death to promote cutaneous melanoma by modulating ULK1 phosphorylation

**DOI:** 10.1038/s41419-021-04362-8

**Published:** 2021-11-10

**Authors:** Shuo Han, Xue Li, Ke Wang, Dingheng Zhu, Bingyao Meng, Jieyu Liu, Xiaoting Liang, Yi Jin, Xingyuan Liu, Qian Wen, Liang Zhou

**Affiliations:** 1 Department of Toxicology, Guangdong Provincial Key Laboratory of Tropical Disease Research, School of Public Health, Southern Medical University, Guangzhou, China; 2 School of Clinical Medicine and Technology, Sichuan Vocational College of Health and Rehabilitation, Zigong, China; 3 Dermatology Hospital, Southern Medical University, Guangzhou, China; 4 Institute of Molecular Immunology, School of Laboratory Medicine and Biotechnology, Southern Medical University, Guangzhou, Guangdong China

**Keywords:** Oncogenes, Macroautophagy, Melanoma

## Abstract

Uncontrolled overactivation of autophagy may lead to autophagic cell death, suppression of which is a pro-survival strategy for tumors. However, mechanisms involving key regulators in modulating autophagic cell death remain poorly defined. Here, we report a novel long noncoding RNA, p53 upregulated regulator of p53 levels (PURPL), functions as an oncogene to promote cell proliferation, colony formation, migration, invasiveness, and inhibits cell death in melanoma cells. Mechanistic studies showed that PURPL promoted mTOR-mediated ULK1 phosphorylation at Ser757 by physical interacting with mTOR and ULK1 to constrain autophagic response to avoid cell death. Loss of PURPL led to AMPK-mediated phosphorylation of ULK1 at Ser555 and Ser317 to over-activate autophagy and induce autophagic cell death. Our results identify PURPL as a key regulator to modulate the activity of autophagy initiation factor ULK1 to repress autophagic cell death in melanoma and may represent a potential intervention target for melanoma therapy.

## Introduction

Cutaneous melanoma (CM, hereafter named as melanoma) is one of the most malignant human tumors. The global incidence of melanoma is ~15–25 cases per 100,000, with an annual growth rate of 3–5% [1]. Chronic ultraviolet exposure acts as the prominent risk factor for the malignant transformation of melanocytes to melanoma [1]. Despite the advances of clinical diagnosis and treatment to lower the mortality, high risk of relapse or distant metastasis of late stage melanoma is still associated with poor prognosis and relative short survival period (6–12 months) [1]. Understanding the molecular mechanisms used by melanoma to oppose stresses and maintain homeostasis is still a big challenge and need further in-depth exploration.

During the past three decades, one of the big challenges of clinical oncologists was to develop efficient therapy to eliminate cancer cells by inducing programmed cell death (PCD) [2]. In mammalian cells, PCD is morphologically classified into three major types: type I cell death (Apoptosis), type II cell death (Autophagic cell death), and type III cell death (Necrosis) [3]. Apoptosis is characterized by a series of distinct morphologic and biochemical changes, including cell shrinkage, nuclear aggregation, chromatin condensation, formation of apoptotic bodies [3], while necrosis is featured by passive energy-independent cell death and induced by extreme physicochemical stresses [3]. Autophagic cell death (ACD) is characterized by the formation of typical vacuoles, focal plasma membrane disruption, focal nuclear concavity, and even loss of cellular organelles with fragmented ER and electron-dense mitochondria, which are induced by over-activated autophagic flux [3–5]. Specially, ACD could be completely repressed by inhibition of the autophagy pathway [3].

For tumor cells, their daily task is to deal with the body’s natural defense system and various therapeutic treatments, which contributes to their powerful vitality [6]. However, the knowledge about how tumor cells execute the death-suppressive function is still limited, especially for autophagic cell death. ATG genes apparently plays roles in regulating autophagic cell death. In ovarian cancer, depletion of ATG5 or ATG7 gene prevented H-Ras induced autophagic cell death [7]. Na^+^/K^+^-ATPase has been demonstrated to be involved in one form of BECN1-derived peptide- or starvation-induced autophagic cell death, which could be prevented by knockdown of ATG13 or ATG14 [8]. Loss of ATG4B, ATG7, and ATG12 decreased resveratrol-induced LC3 lipidation and enhanced cell survivability [9]. RNA molecules were hardly shown to be involved in autophagic cell death. miR-14 has been shown to target IP3K2 to modulate IP3 signaling and further calcium release from ER to connect with development-related autophagic cell death [10]. More functional RNA molecules in autophagic cell death still need to be explored.

Long noncoding RNAs (LncRNAs) are a group of transcripts with length exceeding 200 nucleotides and play diverse roles in pathophysiological processes including carcinogenesis [11]. LncRNAs could act as a recruiter, decoy, or scaffold to transcriptionally regulate gene expression or a sponge to modulate miRNA function [12]. Undoubtedly, LncRNAs are important players in melanoma development [13]. For example, PANDAR could act as an oncogene to promote cell proliferation, migration and invasion in melanoma cells and facilitates epithelial-mesenchymal transition [14]. SAMMSON was highly expressed in 90% of human melanoma and could bind with p32 to enhance its mitochondrial localization and promote the proliferation and survivability under stresses [15]. Although great progresses have been made in investigating the function and mechanism of LncRNAs, there is still a long way to go before we fully understand the role of LncRNAs, especially in relation with cell death and further autophagic cell death.

In this study, we identified ‘p53 upregulated regulator of p53 levels’ (PURPL) is highly expressed in melanoma and functions as an oncogene in promoting the proliferation, colony formation, migration, and invasiveness in melanoma cells by suppressing cell death. To explore the underlying mechanism, RNA pulldown followed by high-performance liquid chromatography-mass spectrometry (HPLC-MS) analysis was performed and indicated that PURPL physically associates with mTOR to directly regulate the differential phosphorylation of ULK1 to suppress autophagic cell death and maintain the survivability of melanoma cells. The anti-autophagic and pro-survival roles of PURPL were further validated by in vitro and in vivo assays. Our findings highlight a novel oncogenic role of PURPL to suppress autophagic cell death mediated by regulating differential ULK1 phosphorylation in melanoma, which may provide novel intervention targets for melanoma therapy.

## Results

### PURPL is highly upregulated in melanoma cells and tumors

**Fig. 1:**
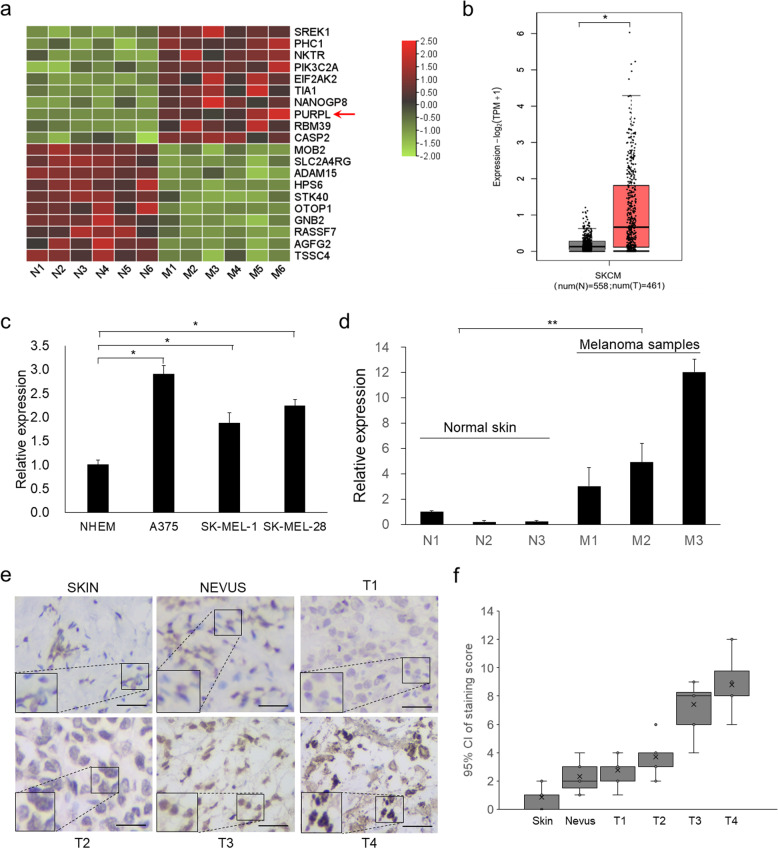
PURPL is upregulated in melanoma cells and primary tumors and acts as oncogene. **a** The top 10 upregulated and top 10 downregulated genes in primary melanomas were sorted according to the deviation values and shown in a heat map. Color bars on the right represent ranges of *z* value. PURPL was indicated by a red arrow. **b** The expression of PURPL in melanoma (461 cases) compared with normal tissues (558 cases). The original data is from The Cancer Genome Atlas Program (TCGA) database and analyzed by Gene Expression Profiling Interactive Analysis (GEPIA, http://gepia.cancer-pku.cn/). The expression levels of PURPL were detected by qPCR in **c** primary normal human epidermal melanocytes (NHEM) and melanoma cell lines (A375, SK-MEL-1, and SK-MEL-28) or **d** normal skin tissues and melanomas. The qPCR data represent the average of three independent experiments ±s.d. **e** in situ hybridization (ISH) detection of PURPL on paraffin sections of melanoma and normal skin specimens. Representative images with various levels of staining (negative or weak from normal tissues, strong from tumor tissues) are shown. Scale bar: 100 µm. **f** Association of PURPL staining scores with tumor grade (Normal skin tissues, Nevi, T1, T2, T3, and T4). Data are plotted as the means of 95% confidence interval ±s.d. *P < 0.05, **P < 0.01, ***P < 0.001.

To explore LncRNAs highly expressed in melanoma, we sorted the public transcriptomic data [16] after z-score standardization [17]. One of the top ten highest-expressed genes is PURPL which is ranked as the eighth highest-expressed gene and the highest-expressed LncRNA in melanoma (Fig. 1a). We also checked PURPL expression in The Cancer Genome Atlas Program (TCGA) database and confirmed the higher expression levels of PURPL in melanoma group compared with normal group (Fig. 1b). The higher expression of PURPL was practically validated by examining the expression levels of PURPL in the melanoma cell lines (A375, SK-MEL-1 and SK-MEL-28) compared with the primary normal human epidermal melanocytes (NHEM) (Fig. 1c). To extend this analysis to clinic samples, we collected and verified higher expression of PURPL in melanoma tumors compared with normal skins (Fig. 1d). Importantly, PURPL expression was further examined in paraffin-embedded sections of 52 melanoma and 10 normal skin specimens by in situ hybridization (ISH). As predicted, higher expression of PURPL (stronger staining) can be detected in all melanoma tumors examined (Fig. 1e). Further scoring of PURPL staining showed a positive correlation with ascending melanoma grade. Specifically, an evident increasing trend was observed across from normal skin tissue, nevi, the early-stages of melanoma (Clark I and II) (*P* < 0.05) to late-stages of melanoma (Clark III & IV) (*P* < 0.05) (Fig. 1f). Collectively, the above results suggested that PURPL is upregulated in melanoma cells and primary melanoma tumors, and is positively correlated with the Clark staging of melanoma.

### PURPL promotes melanoma cell proliferation, colony formation, migration, and invasiveness

**Fig. 2:**
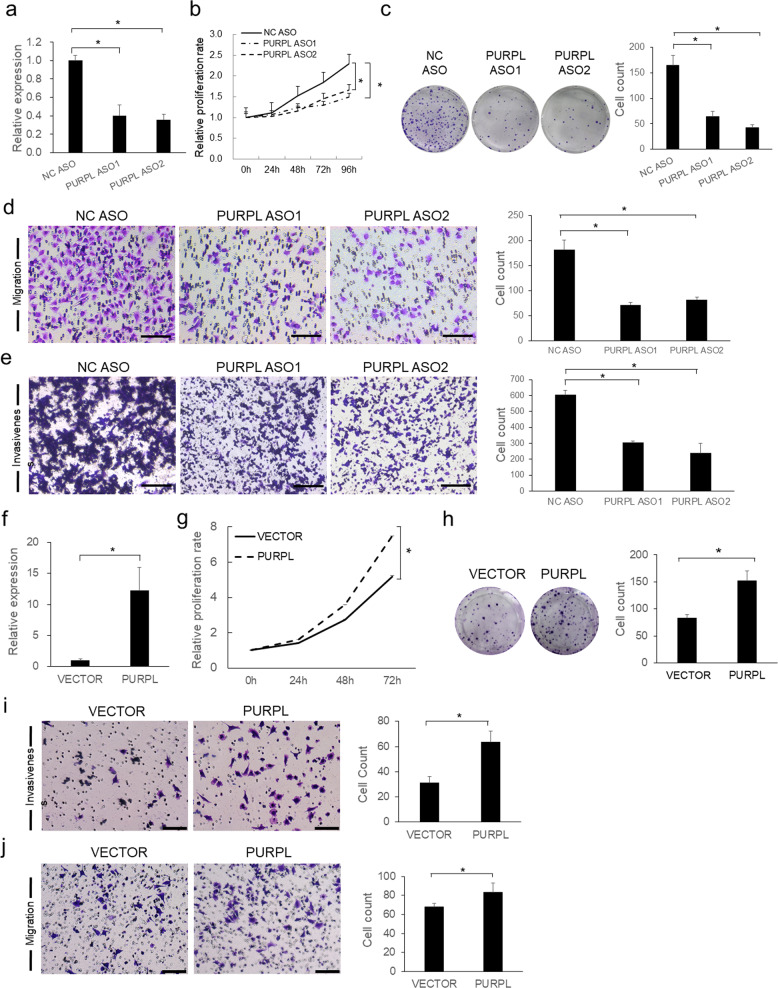
PURPL promotes cell proliferation, migration, and invasiveness in melanoma cells. **a** PURPL RNA expression was detected by qPCR after depletion of PURPL by ASOs in A375 cells. Measurement of cell proliferation by CCK-8 assay (**b**), colony formation assay (**c**), transwell migration assay (**d**), and matrigel invasiveness measurement (**e**) were performed in A375 cells treated with ASOs targeting PURPL. **f** PURPL RNA expression was detected by qPCR after overexpression of PURPL in A375 cells. Measurements of cell proliferation by CCK-8 assay (**g**), colony formation assay (**h**), transwell migration assay (**i**), and matrigel invasiveness measurement (**j**) were performed in A375 cells overexpressing PURPL. Scale bar: 100 µm. One-way ANOVA and Dunnett’s multiple comparison test. Means ± s.d. *P < 0.05, **P < 0.01, ***P < 0.001.

The expression levels of PURPL are varied in different types of cancer compared with corresponding normal tissues (Supplementary Fig. S1), which may indicate its diverse roles in different cancers. The upregulation of PURPL in melanoma prompted that PURPL may have an oncogenic role in melanoma development. To test this hypothesis, depletion of PURPL was achieved by transfecting antisense oligonucleotides (ASOs) against PURPL into melanoma cells. Significant loss of PURPL (Fig. 2a and Supplementary Fig. S2a and S3a) led to a drastic decrease of proliferation capacity in A375 (Fig. 2b), SK-MEL-28 (Supplementary Fig. S2b), and SK-MEL-1 (Supplementary Fig. S3b) cells, which was also supported by the colony formation assay showing significantly less numbers of colonies formed in PURPL ASO-treated groups than control group (Fig. 2c). The mobility of A375 cells was significantly decreased upon PURPL knockdown as indicated by Transwell migration assay, demonstrating significantly less cells penetrated the pores of the membrane than NC ASO-treated group (Fig. 2d). Matrigel invasiveness measurement showed that knockdown of PURPL also significantly compromised the invasive capacity of melanoma cells (Fig. 2e). Conversely, overexpression of PURPL in A375 cells promoted melanoma cell proliferation, colony formation, migration, and invasiveness compared with cells transfected with empty vector (Fig. 2f–j).

Together, the above data demonstrated that PURPL acts as oncogene to promote tumor growth, colony formation, migration, and invasiveness in melanoma cells.

### PURPL directly interacts with ULK1 to regulate autophagy

**Fig. 3:**
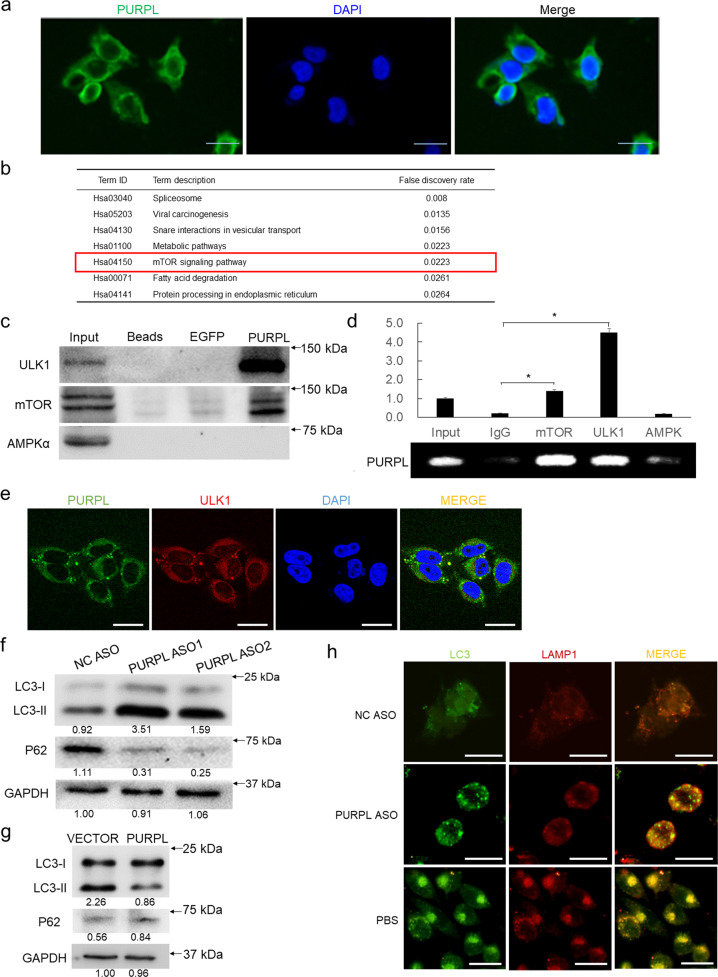
PURPL is mainly localized in cytoplasm and interacts with ULK1 to regulate autophagy. **a** Visualization of PURPL in A375 cells by RNA fluorescence in situ hybridization (FISH). **b** Kyoto Encyclopedia of Genes and Genomes (KEGG) pathway analysis of the proteins interacting with PURPL by subtracting the proteins non-specific binding to EGFP RNA after identified by HPLC-MS. “mTOR signaling pathway” is highlighted. **c** Biotin-labeled PURPL transcript was used to retrieve interacting protein partners by RNA pulldown with beads only and EGFP as controls. The resulting protein mix was applied to detect ULK1, mTOR, and AMPK by western blot. **d** RNA immunoprecipitation (RIP) assay was performed using antibodies against ULK1, mTOR, and AMPK while IgG was used as control. The retrieved PURPL RNA was detected by qPCR. **e** Co-localization of PURPL with ULK1 was detected using FISH and IF staining and observed by confocal microscope. **f**, **g** LC3B and p62 proteins were detected after PURPL knockdown or overexpression by western blot, GAPDH was used as loading control. **h** The autophagy levels in melanoma cells following PURPL knockdown by ASO mix (ASO1:ASO2 in 1:1 ratio) was evaluated by LC3 and LAMP1 staining. PBS-treated melanoma cells were used as positive control for autophagy induction. Scale bar, 50 μM. *p < 0.05, **p < 0.01, ***p < 0.001.

PURPL has been reported to be localized in nucleus and regulate p53 stability in colorectal cancer [18]. Before we start the mechanistic study, the subcellular localization of PURPL in melanoma cells was detected with fluorescent in situ hybridization (FISH) and observed distinct cytoplasmic localization of PURPL, while small portion of PURPL is indeed in nucleus (Fig. 3a). Since PURPL is mainly localized in cytoplasm, the role of PURPL in melanoma should be quite different from the role in colorectal cancer. Seeing the current theory that LncRNAs play roles by associating with proteins [11], RNA pulldown assay was performed using an in vitro*-*transcribed full-length PURPL RNA together with a control EGFP RNA to explore how PURPL knockdown inhibits the proliferation of melanoma. The specific binding partners of PURPL were detected by comparing the differential binding protein profiles between PURPL and EGFP RNAs identified with HPLC-MS (Supplementary Table S1), the list of which were sent for Kyoto Encyclopedia of Genes and Genomes (KEGG) and Gene Ontology (GO) analysis. The clustered KEGG items include “Spliceosome”, “Viral carcinogenesis”, “SNARE interactions in vesicular transport”, “Metabolic pathways”, and “mTOR signaling pathway” (Fig. 3b and Supplementary Table S2).

Two of the proteins clustered in “mTOR signaling pathway” are ULK1 and mTOR definitely identified by HPLC-MS (Supplementary Fig. S4), among which ULK1 is critical for the initiation of autophagy while mTOR is able to phosphorylate ULK1 and inhibit autophagy [19]. To verify the results from HPLC-MS analysis, RNA pulldown was performed and confirmed that PURPL directly interacts with ULK1 and mTOR (Fig. 3c). As AMPK has also been reported to be the interacting partner of ULK1 and regulate autophagy [19], we tested its potential interaction with PURPL by RNA pulldown assay. However, PURPL did not show any interaction with AMPK (Fig. 3c). Further validation by RNA immunoprecipitation (RIP) using specific antibodies confirmed that PURPL interacts with ULK1 and mTOR but not with AMPK (Fig. 3d). We also verified the co-localization of PURPL with ULK1 using FISH and IF staining respectively using confocal microscopy (Fig. 3e). Thus, we hypothesized that PURPL intervenes in autophagy to modulate melanoma progression by directly interacting with ULK1 and mTOR. To verify this hypothesis, the expression levels of PURPL were knocked down using specific ASOs and the autophagy-related features were checked. Depletion of PURPL significantly increased the amount of LC3B-II, reduced the p62 expression (Fig. 3f) and promoted the formation of LC3B foci (Fig. 3h) in melanoma cells, which indicates the enhancement of autophagic flux. At the same time, overexpression of PURPL decreased LC3B-II level and led to p62 accumulation (Fig. 3g).

**Fig. 4:**
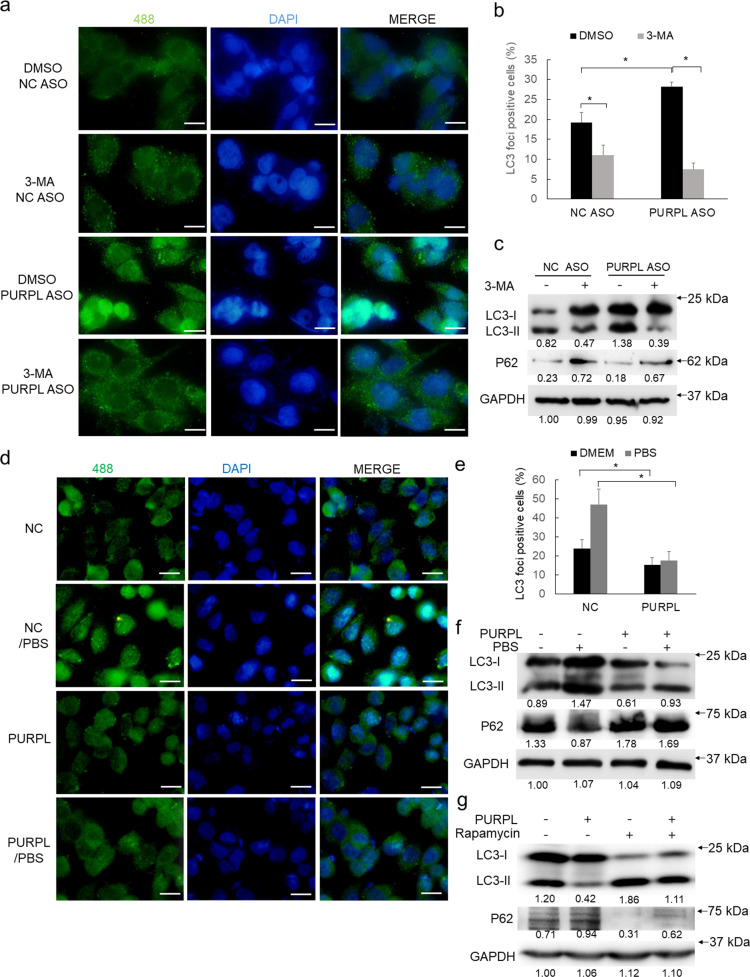
Validation of PURPL’s role in regulating autophagy. **a**, **b** The PURPL-regulated autophagy was validated in autophagy inhibitor 3-MA-treated (5 mM for 5 h) melanoma cells following PURPL knockdown by ASO mix (ASO1:ASO2 in 1:1 ratio) using LC3 staining. **c** LC3B and P62 protein was detected after PURPL knockdown and 3-MA treatment by western blot, GAPDH was used as loading control. **d**, **e** The PURPL-regulated autophagy levels in melanoma cells following PURPL overexpression was evaluated by LC3 staining. PBS-treated melanoma cells were used as positive control for autophagy induction. **f** LC3B and P62 protein was detected after PURPL overexpression and PBS treatment by western blot, GAPDH was used as loading control. **g** LC3B and P62 protein was detected after PURPL overexpression and Rapamycin treatment by western blot, GAPDH was used as loading control. Scale bar, 100 μM. *p < 0.05, **p < 0.01, ***p < 0.001.

To validate if PURPL regulates autophagy, we treated melanoma cells with autophagy inhibitor 3-MA. In addition to the decrease of LC3 foci positive cells in control group, 3-MA treatment could significantly compromise the number of LC3 foci positive cells induced by PURPL depletion (Fig. 4a, b). The highly upregulated amount of LC3B-II was also consistently repressed by 3-MA treatment (Fig. 4c). Further, overexpression of PURPL significantly inhibited the formation of LC3 foci induced by PBS treatment-mediated starvation (Fig. 4d, e), which is consistent with the less generation of LC3B-II in PURPL-overexpressed groups (Fig. 4f). Rapamycin treatment-induced autophagy could also be suppressed by PURPL overexpression (Fig. 4g). Collectively, PURPL interacts with ULK1 and negatively regulates autophagy.

### Knockdown of PURPL induces autophagic cell death

**Fig. 5:**
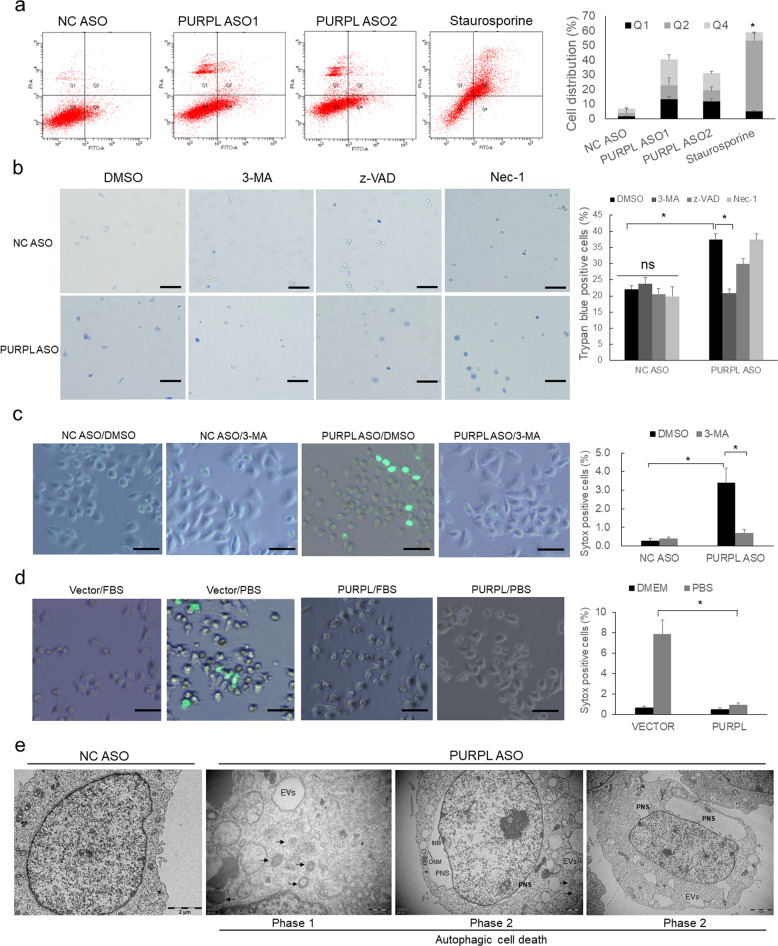
PURPL functions in repressing autophagic cell death. **a** The cell distribution was detected by flow cytometry with Annexin V/PI double staining in A375 melanoma cells after depletion of PURPL. Cells treated with 200 nM Staurosporine for 24 h were used as apoptosis control. **b** Trypan blue staining was performed to detect the major cell death type induced by PURPL depletion in melanoma cells with 3-MA (5 mM for 5 h), zVAD (100 μM for 24 h), and Nec-1 (50 μM for 24 h) treatments. Scale bar, 100 μM. **c** Sytox green staining was performed to evaluate the effects of 3-MA treatment on autophagic cell death induced by PURPL depletion. Scale bar, 100 μM. **d** Sytox green staining was performed to evaluate the role of PURPL in repressing starvation-induced autophagy in melanoma cells. PBS-treated melanoma cells were used as positive control for autophagy induction. Scale bar, 100 μM. **e** Autophagic cell death was detected by transmission electron microscopy (TEM). Arrows indicates autophagosomes. Typical features of autophagic cell death includes the formation of empty vacuoles (EVs) and perinuclear space (PNS) by the separation of outer nuclear membrane (ONM) and inner nuclear membrane (INM). Scale bar, 2 μM. One-way ANOVA and Dunnett’s multiple comparison test. Means ± s.d., *p < 0.05, **p < 0.01, ***p < 0.001.

The role of autophagy could be classified as pro-survival autophagy and anti-survival autophagic cell death. As the tumor-suppressive effect after PURPL depletion may indicate PURPL regulates one of the above two types of autophagy, we performed fluorescence-activated cell sorting (FACS) analysis with Annexin V-FITC/PI double staining and found that a large portion of cells are clustered in the first quadrant, which is quite different from the death induced by apoptotic inducer staurosporine in A375 cells (Fig. 5a) and in SK-MEL-28 cells (Supplementary Fig. S2e). Considering PURPL can regulate autophagy, we hypothesized that PURPL may negatively modulate autophagic cell death. To discriminate which type of cell death is regulated by PURPL, melanoma cells with PURPL knockdown were treated with different cell death inhibitor including 3-MA (autophagic cell death), z-VAD (apoptosis), and Nec-1 (necrosis), and observed with Trypan blue staining. Trypan blue exclusion assay showed that knockdown of PURPL induced significant increase of cell death rate while 3-MA treatment almost fully compromised the induction of cell death compared with control group (Fig. 5b). Such results indicated that PURPL functions mainly in protecting against autophagic cell death. Further, 3-MA treatment diminished the autophagic cell death induced by loss of PURPL using Sytox Green (a nucleic dye excluded by live cells) staining (Fig. 5c). Further, overexpression of PURPL could eliminate the drastic cell death induced by starvation (PBS treatment) (Fig. 5d). Especially, loss of PURPL significantly induced the formation of numerous autophagosomes, empty vacuoles (Phase 1), and perinuclear space (PNS) by the separation of outer nuclear membrane (ONM) and inner nuclear membrane (INM) (Phase 2) detected by transmission electron microscope (TEM) analysis (Fig. 5e), which are the direct evidences for autophagic cell death [5]. Collectively, PURPL plays roles in repressing autophagic cell death.

### PUPRL differentially regulates the phosphorylation of ULK1 to repress autophagic cell death

**Fig. 6:**
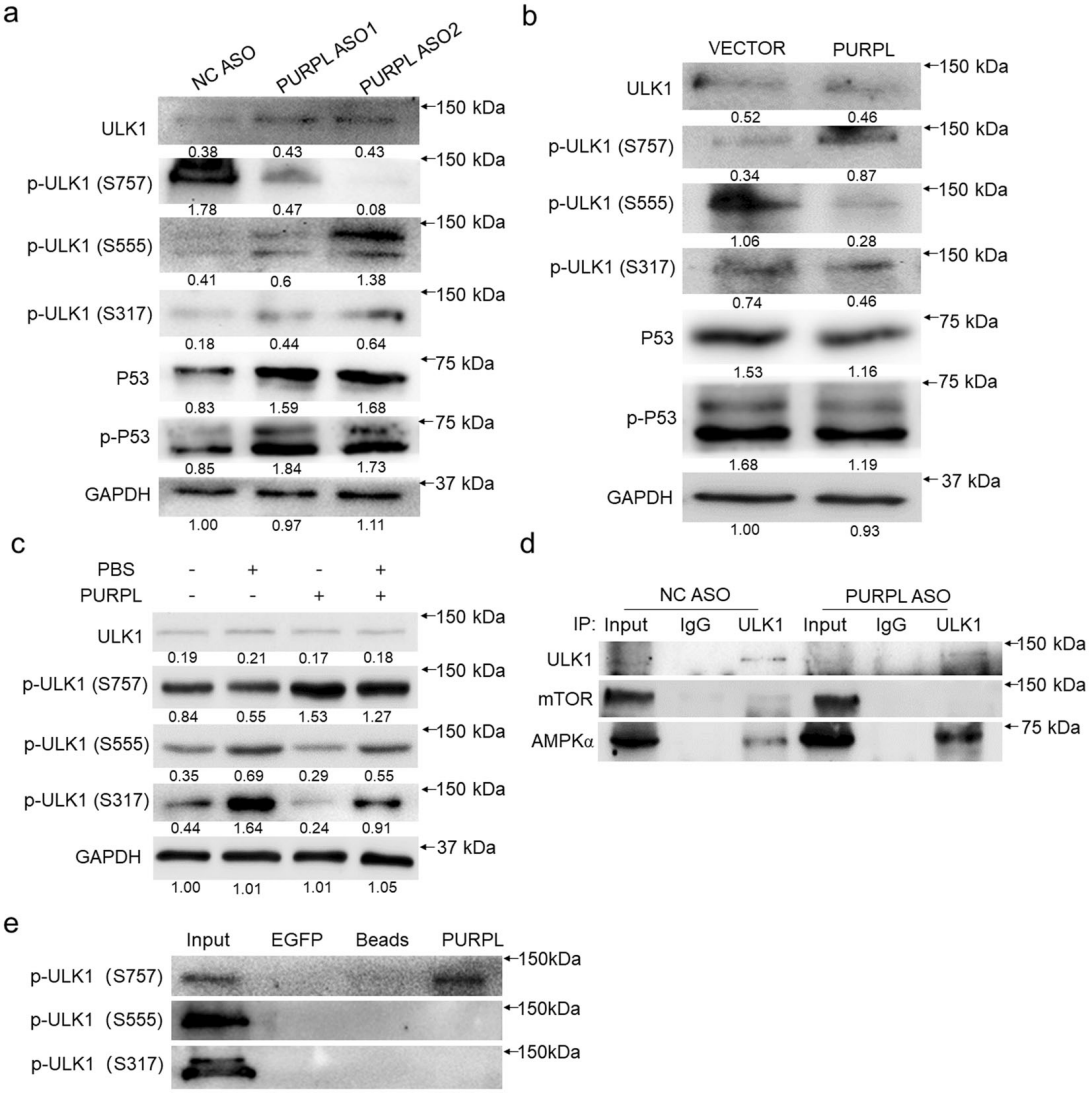
PURPL interacts with mTOR and ULK1 to promote mTOR-mediated anti-autophagic phosphorylation of ULK1. **a**, **b** The phosphorylation status of ULK1 was detected using phosphorylation site-specific antibodies against Ser757, Ser555, and S317 in response to PURPL knockdown or overexpression, while P53 and p-P53 were also detected by western blot. ULK1 and GAPDH were also detected as controls respectively. **c** ULK1 phosphorylations were detected using the above site-specific antibodies to evaluate the role of PURPL in repressing starvation-induced autophagy in melanoma cells. PBS-treated melanoma cells were used as positive control for autophagy induction. **d** Immunoprecipitation of ULK1 was performed to check if loss of PURPL influences the interactions of ULK1 with mTOR or AMPK. **e** The interactions of PURPL with various ULK1 phosphorylations were detected by RNA pulldown.

Direct phosphorylation of ULK1 by AMPK or mTOR determines distinct functions of ULK1 in autophagy [19]. mTOR-mediated phosphorylation of ULK1 at Ser757 can inactivate autophagy while AMPK-mediated phosphorylations of ULK1 at Ser317 and Ser555 function in activating or even over-activating autophagy [19, 20]. Since we have already demonstrated PURPL interacts with mTOR but not with AMPK (Fig. 3c, d), we next probed how such relationship influences ULK1 phosphorylations status using site-specific phosphorylation antibodies. Depletion of PURPL strongly repressed the amount of p-ULK1 (Ser757) while enhancing p-ULK1 (Ser555 and Ser317) generation in A375 (Fig. 6a), SK-MEL-28 (Supplementary Fig. S2f), and SK-MEL-1 (Supplementary Fig. S3e) cells. At the same time, overexpression of PURPL produced the inverse effects by upregulating p-ULK1 (Ser757) and inhibiting p-ULK1 (Ser555 and Ser317) in A375 (Fig. 6b) and SK-MEL-28 (Supplementary Fig. S2g) cells. We also checked the expression levels and activities of P53 in response to the change of PURPL levels by detecting total P53 and phosphorylated P53. The results showed that PURPL negatively regulates P53 expression and activity (Fig. 6a, b). To verify the above differential effects of PURPL to ULK1 phosphorylation, the melanoma cells were starved by PBS treatment to induce autophagy and checked the ULK1 phosphorylation status. PBS treatment decreased p-ULK1 (Ser757) amount and enhanced p-ULK1 (Ser555 and Ser317) formation, while overexpression of PURPL acted inversely to promote p-ULK1 (Ser757) production and repressed p-ULK1 (Ser555 and Ser317) formation (Fig. 6c). To detect if PURPL regulates ULK1 interaction with mTOR or AMPK, we knocked down the expression of PURPL and performed ULK1 immunoprecipitation. The results showed that PURPL depletion interfered the interaction between ULK1 and mTOR while promoted ULK1 and AMPK interaction (Fig. 6d). Such finding is also supported by the direct interaction between PURPL and p-ULK1 (Ser757) but not with p-ULK1 (Ser555) or (Ser317), which is consistent with the anti-autophagic effects in the presence of PURPL (Fig. 6e). Collectively, PURPL inhibits autophagy by promoting the formation of mTOR-mediated anti-autophagic p-ULK1 (Ser757) and repressing AMPK-mediated pro-autophagic p-ULK1 (Ser555 or Ser317).

### Loss of PURPL enhances autophagic cell death and compromise the proliferation of melanoma in vivo

**Fig. 7:**
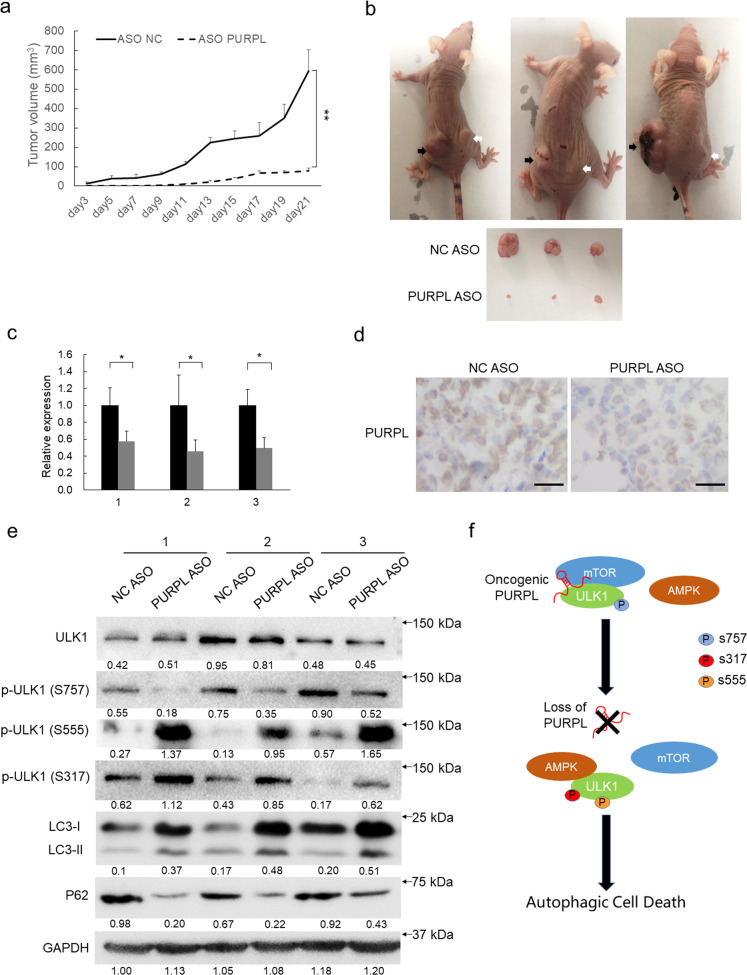
Loss of PURPL activates autophagic cell death and represses tumor growth in vivo. **a** Loss of PURPL inhibits subcutaneous melanoma growth in a mouse xenograft model. Tumor volumes (mm^3^) were plotted according to day. The statistical data represent the average of three independent experiments ±s.d, respectively. **b** The mice were sacrificed at the end of the experiment and the dissected tumors from three representative mice are shown. black arrows indicate the NC ASO-treated xenografts whereas white arrows indicate PURPL ASO-treated xenografts. **c** PURPL expression was measured in the dissected tumors by qPCR. The statistical data represent the average of three independent experiments ±s.d. **d** The expression levels of PURPL in tumor sections were evaluated using in situ hybridization. Scale bar, 100 µm. **e** Protein amount of total ULK1, phosphorylated ULK1, LC3B, and p62 was detected in paired control and PURPL-depleted groups by western blot, GAPDH was used as loading control. **f** A model depicts PURPL interacts with mTOR and ULK1 to differentially regulate ULK1 phosphorylation to repress autophagic cell death and promote melanoma development.

To evaluate the pro-carcinogenic and anti-autophagic function of PURPL in vivo, a xenograft tumor model was established in immunocompromised mice. No significant difference in tumor size was initially apparent between the control group and PURPL knockdown group. After the 9th day, PURPL depletion markedly inhibited tumor growth compared with the control group and led to evidently smaller tumor mass at the end of the evaluation period (Fig. 7a, b). Loss of PURPL expression in the PURPL depletion group was confirmed by qPCR and ISH staining (Fig. 7c, d). Furthermore, PURPL depletion led to the significant upregulation of LC3B-II, less p62 amount, inhibition of p-ULK1 (Ser757) formation and enhancement of p-ULK (Ser555) and (Ser317) generation (Fig. 7e).

Collectively, the in vivo experiments indicated that PURPL functions in repressing autophagic cell death to promote melanoma growth by differentially modulating ULK1 phosphorylations.

## Discussion

As a catabolic process, autophagy serves as a low-level constitutive and protective mechanism by degrading cellular contents within lysosomes for efficiently recycling useful materials, although hyperactivation of autophagy could lead to autophagic cell death [4, 21]. However, there are still mysteries need to be deciphered, especially in melanoma, a cancer type with less explorations in autophagy.

Autophagy is driven by more than 30 well-conserved ATG proteins in metazoan. Many signaling pathways are involved in fine-tunning the autophagy, including phosphatidylinositol 3-kinase (PI3K), Adenosine 5′-monophosphate-activated protein kinase (AMPK), and mammalian target of rapamycin (mTOR) pathways [21]. LncRNAs actively take part in the regulation of autophagy by regulating the above important pathways. For example, LncRNA H19 induces autophagic cell death in cerebral ischemia and reperfusion (I/R) injury by inhibiting DUSP5 to phosphorylate ERK1/2 and provoke autophagy [22]. NBR2 (the lncRNA neighbor of BRCA1 gene 2) functions in binding to AMPK and promoting its activation, while AMPK activation under energy stress induced NBR2 expression. So, AMPK/NBR forms a feed-forward loop to sustain AMPK activation in response to energy stress in normal cells [23]. In atherosclerosis, lncRNA-FA2H-2 transcriptionally inhibited MLKL expression and loss of lncRNA-FA2H-2 led to the activation of inflammation and inhibition of autophagic flux which is dependent on mTOR pathway [24].

ULK1 (ATG1) is a serine/threonine kinase essential for autophagy initiation. The activity of ULK1 in autophagy is regulated by mTOR and AMPK, which generate differential phosphorylations by physical interaction with ULK1 and producing either pro-autophagic phosphorylations (Ser317 and Ser555) by AMPK or anti-autophagic phosphorylation (Ser757) by mTOR [19, 20, 25]. LncRNAs have been shown to regulate the expression or activity of ULK1. Risa is a cytoplasmic poly(A+) RNA and regulates insulin sensitivity in mice. Depletion of Risa enhanced the phosphorylation of Ulk1 (Ser757) which contributed to autophagy initiation. However, Ulk1 (Ser757) is used as biomarker for autophagy status and no further mechanism was shown about if Risa could directly regulate Ulk1 phosphorylation [26]. PTENP1 is a pseudogene of known tumor suppressor PTEN and could decoy oncomiR including miR-17 and miR-20a to indirectly regulates ULK1 expression [27]. Similarly, LncRNAs, including PVT1, LUCAT1, SNHG6, and MALAT1, could indirectly regulate ULK1 expression through acting as microRNA sponge to regulate autophagy [28–31]. However, the direct regulation of ULK1 phosphorylation by LncRNAs has been hardly explored.

Recently, LncRNAs have been reported to regulate protein modifications in addition to the interactions with their partners. Lnc-DC directly bound to STAT3 to promote its phosphorylation at Tyr705 by preventing STAT3 from interaction with SHP1 and dephosphorylation by SHP1 [32]. LINK-A (long intergenic noncoding RNA for kinase activation) was highly upregulated in triple-negative breast cancer (TNBC) and functioned in facilitating BRK kinase activation which promoted HIF-1α Tyr565 phosphorylation and stabilized HIF-1α by interfering with Pro564 hydroxylation to enhance HIF-1α-p300 interaction and HIF-1α transcriptional activity under normoxic condition [33]. LINK-A mediated AKT and Phosphatidylinositol-3,4,5-trisphosphate (PIP3) interaction, which led to the hyperactivation of AKT to promote tumorigenesis and resistance to AKT inhibitors [34]. In this study, we showed PURPL is actively involved in regulating ULK1 activity during autophagy initiation. Depletion of PURPL inhibits ULK1 phosphorylation at Ser757 and promotes ULK1 phosphorylation at Ser317 and Ser555, which leads to the induction of autophagic cell death. We pioneeringly provide the evidences to support the role of LncRNA in physical interaction with ULK1 and regulating its modifications. The involvement of LncRNAs in the modifications of proteins is quite interesting, which naturally demonstrates the depth and extent of LncRNAs participating in pathophysiologic processes.

Collectively, our study showed PURPL is highly expressed in melanoma and its subcellular localization is mainly in cytoplasm, which is drastically different compared from previous report [18] and drives us to explore the role of PURPL in cytoplasm. PURPL functions as an oncogene by promoting the proliferation, colony formation, migration, and invasiveness of melanoma cells. Importantly, loss of PURPL induces autophagic cell death. Mechanistic study showed that PURPL physically interacts with ULK1 and differentially regulates its phosphorylation to suppress autophagic cell death to maintain the survivability of melanoma cells (Fig. 7f). Our findings highlight the oncogenic and autophagy-suppressive roles of PURPL in melanoma and emphasize a novel mechanism to repress autophagic cell death, which may provide novel intervention targets for melanoma therapy.

## Materials and methods

### Patient samples

Melanoma samples were obtained from patients diagnosed with cutaneous melanoma from June 2018 to October 2020 in the Dermatology Hospital affiliated to Southern Medical University and Guangzhou KingMed Diagnostics. Briefly, a total of 52 specimens of melanoma (31 samples from men and 21 from women) and 10 specimens from normal skin were included in this study. Biopsies obtained during surgery were immediately frozen using liquid nitrogen and subdivided for subsequent RNA, protein extraction and paraffin embedding. Tumors were classified according to the American Joint Committee on Cancer classification of melanoma (eighth edition) [35]. This study was approved by the Institutional Review Board of Nanfang Hospital affiliated to Southern Medical University. All investigations complied to the principles of the Declaration of Helsinki.

### Animal studies

Male athymic nude mice (BALB/C-nu/nu, 4–5 weeks old) purchased from the animal center of Southern Medical University were used for xenograft studies. The mice were euthanized by cervical dislocation to prevent suffering. This study was approved by the Institutional Animal Care and Use Committee (IACUC) of Southern Medical University (Approval code L2019178). They are in accordance with the guidelines of the Asian Federation of Laboratory Animal Science Associations (AFLAS) and the National Regulations for the Administration of Affairs Concerning Experimental Animals (8 January 2011). Mouse transportation, housing, and breeding were conducted according to the recommendations of “The use of non-human animals in research”.

### Cell lines

Melanoma lines A375 (Female, Guangzhou Cellcook Biotech Co., Ltd) SK-MEL-28 (Male, Shanghai Xinyu Biological Technology Co., Ltd.) and SK-MEL-1 (Male, Procell Life Science & Technology Co., Ltd.), Human Epidermal Melanocytes (Cell Systems) were cultured in Dulbecco’s modified Eagle medium (DMEM, Life Technologies) supplemented with 10% fetal bovine serum (ExCell Bio, FSP500) or Complete Classic Medium with Serum and CultureBoost™ (4Z0-500) and maintained at 37 °C with 5% CO_2_ in a humidified atmosphere. The STR profiles authentication information of all the cell lines used in this study were listed in Supplementary Fig. S5–S7. All the cell lines have been tested and shown to be no mycoplasma contamination.

### RNA isolation and quantitative real-time PCR

Total RNAs from cells were extracted using TransZol reagent (TransGen Biotech Co., Ltd.) according to the manufacturer’s instructions. cDNAs were prepared using EasyScript All-in-One First-Strand cDNA Synthesis SuperMix for qPCR (One-Step gDNA Removal) (TransGen Biotech, AE341). mRNA expression analysis was performed using PerfectStart Green qPCR SuperMix (TransGen Biotech, AQ601) on a LightCycler 96 Detection System (Roche) using *GAPDH* for normalization. Primers used in this study are listed in Supplementary Table S3.

### RNA-Seq data analysis

Normalized transcriptomic data from biopsies of primary melanomas samples (<57) and benign melanocytic nevi (*n* = 23) were downloaded from the GEO (http://www.ncbi.nlm.nih.gov/geo), accession GSE112509. The obtained data was calculated with *z*-score (standard score) following the formula: *z*-score = (*X*−*µ*)/*σ* (*X* = standardized random variable, *µ* = sample mean, *σ* = sample standard deviation). All the genes were sorted according to the deviation values calculated by subtracting the average *z* value in normal skin tissues from the average *z* value in melanomas, which were used to generate a heat map containing the top 10 upregulated and top 10 downregulated genes.

### DNA constructs

The PURPL expressing construct was purchased from YouBio Biological Technology Co., Ltd. (http://www.youbio.cn) with sequencing verification.

### Immunoblotting and IHC assays

Total cell extracts were prepared and assayed by western blot as previously described [36–38]. The following primary antibodies and dilutions were used: ULK1 (Cell Signaling Technology, 8054, 1:2000), Phospho-ULK1 (Ser757) (Cell Signaling Technology, 14202, 1:2000), Phospho-ULK1 (Ser317) (Cell Signaling Technology, 37762, 1:2000), Phospho-ULK1 (Ser555) (Cell Signaling Technology, 5869, 1:2000), LC3B (SellckChem, A5202, 1:2000), mTOR (Santa Cruz Biotechnology, sc-517464, 1:2000), P62/SQSTM1 (SellckChem, A5180, 1:2000), P53 (Santa Cruz Biotechnology, sc-47698, 1:2000), p-P53 (Ser315) (WanLeiBio, WLP1333, 1:2000), and GAPDH (Santa Cruz Biotechnology, sc-25778,1:5000). The following secondary antibodies were also used: anti-mouse IgG-horseradish peroxidase (HRP), anti-rabbit IgG-HRP, and anti-goat IgG-HRP (Santa Cruz Biotechnology). Bound antibodies were visualized with the Luminata Forte Western HRP substrate (Millipore).

### In situ hybridization and fluorescence in situ hybridization

Antisense single-stranded DNA probe (Supplementary Table S3) was synthesized and end-labeled with digoxigenin (DIG) (Roche). ISH or FISH was performed in formalin-fixed paraffin-embedded melanoma sections or slides covered with cultured melanoma cells. The pre-hybridization, hybridization, anti-DIG-HRP IgG fraction monoclonal (Jackson, 200-032-156) incubation (1:200), and stained with DAB (Servicebio, G1211) was performed as described in previous studies [38, 39]. Stained ISH or FISH sections were imaged with an Olympus FV1000 Confocal Laser Scanning Microscope or a ZEISS Axio Vert.A1 microscope and at least 10 representative images were collected for statistical analysis. The ISH or FISH staining was performed “blind” with respect to the different treatments.

### Transmission electron microscopy

The treated cells were fixed overnight at 4 °C in 2.5% glutaraldehyde. In next day, the samples were rinsed with phosphate-buffered saline (PBS) and fixed in 1% osmium acid for 1–2 h followed with one more rinse. The fixed samples were dehydrated with a serial concentrations of ethanol solutions (50%, 70%, 80%, 95%). The samples were incubated with the mixed solution of acetone and embedding agent for 1 h, and then transferred into pure embedding agent overnight. Finally, the embedded samples ultra-thinly sectioned and observed with transmission electron microscope Hitachi H-7500.

### RNA immunoprecipitation assay

RIP was performed as described [38, 39]. In all, 5 μg antibodies against ULK1, mTOR, AMPKα, or isotype IgG (Merck Millipore) as a negative control was used.

### RNA pulldown

RNA pulldown was performed as previous described [38, 39]. Biotinylated PURPL transcript and EGFP RNAs were transcribed using a MAXIscript T7/T3 in vitro transcription kit (Ambion) and Biotin RNA labelling Mix (Roche).

### High-performance liquid chromatography-mass spectrometry analysis

A 20-μg sample of immunoprecipitated protein mix was separated by sodium dodecyl sulfate-polyacrylamide gel electrophoresis (SDS-PAGE) and stained with Coomassie brilliant blue R250 and then processed with the Trypsin Profile IGD Kit (Sigma, PP0100). The resulting digest was treated with ZipTip C18 (Merck Millipore, ZTC18S096) then subjected to analysis by Thermo Fisher Scientific orbitrap fusion LC-MS/MS in positive ion, linear, delayed-extraction mode. Calibration was carried out using a standard peptide mixture. The mass spectra were subjected to sequence database for searching with Proteome Discoverer v2.1 software (Thermo Scientific).

### Xenograft mouse model

Briefly, 1.0 × 10^7^ cells were subcutaneously implanted into the left and right flanks of male athymic nude mice (BALB/C-nu/nu, 4–5 weeks old). At 8 days after implantation, NC ASO or PURPL ASO oligos were injected into the left or right tumor, respectively; and the injection was repeated every four days. The experiments were performed “blind” with respect to different treatments. Oligos were prepared by pre-incubating 3 nM per mouse with Lipofectamine 2000 (Life Technologies) for 15 min; injections were made using a final volume of 50 μl in serum-free DMEM. The tumor diameters were measured and recorded every 2 days to generate a tumor growth curve. After tumor growth assessment, the tumors were excised and separated with snap-frozen for RNA and protein extraction or paraffin-embedded for ISH detection.

### Cell proliferation and colony forming assays

A375 cells (4,000 per well) cultivated on 96-well plates were transfected with ASOs and cell proliferation was detected after 0, 24, 48, and 72 h using a cell counting kit (TransGen Biotech, FP101) at 450 nm as described in the manual. For the colony forming assay, transfected cells were incubated in six-well plates at 1000 cells per well, which were maintained in DMEM and medium was replaced twice. At day 7, plates were collected after being washed twice with PBS and fixed in 4% paraformaldehyde for 30 min. Finally, the cells were stained with 0.1% crystal violet. Visible colonies were photographed and counted.

### Flow cytometry assay

A375 cells were seeded on a 60-mm dish and transfected with NC ASO, PURPL ASO1, or PURPL ASO2 and cultured for 48 h. TransDetect Annexin V-FITC/PI cell apoptosis detection kit (TransGen Biotech) was applied according to instructions. Cell death was detected and quantified using a Guava easyCyte Flow Cytometry System (Merk Millipore).

### Treatments with inhibitors

Autophagy inhibitor 3-Methyladenine (3-MA) (5 mM, Selleck) and caspase inhibitor Z-VAD-FMK (100 μM, Selleck) treatment was performed in melanoma cells for 5 h after PURPL knockdown or overexpression, while Staurosporine (200 nM, MedChemExpress), Rapamycin (100 μM, Selleck) and Necrostatin-1 (Nec-1, 50 μM, Selleck) was treated for 24 h.

### Transwell assay

To assess cell migration, 2.0 × 10^5^ A375 cells transfected with NC ASO or PURPL ASO were seeded into the 8-μm upper chambers of 12-well plates (Merk Millipore) in serum-free DMEM. During culture at 37 °C for 48 h, the cells in the upper chambers were attracted by the culture medium in the lower chamber, through chemoattractant provided by the included 10% fetal bovine serum. The chambers were washed with PBS twice and fixed with 3.7% formaldehyde. Cells were permeabilized using 100% methanol at room temperature, stained with 0.1% crystal violet, and observed using a microscope after the cells remained in the wells being scraped off with cotton swabs.

### Matrigel invasiveness assay

For the assessment of invasive ability, Matrigel-coated chambers (Merck Millipore) were used to culture transfected A375 cells, of which 1.0 × 10^5^ cells were seeded into the upper chambers. Other treatments were performed as in the migration assay.

### Statistical analysis

Statistical tests were performed for independent-samples with an unpaired *t*-test or one-way ANOVA tests (SPSS version 17.0, SPSS Inc.). All statistical tests incorporated two-tailed tests and homogeneity of variance tests, and were considered to reflect significant differences if *P < 0.05, **P < 0.01, or ***P < 0.001. Details of statistical analyses including sample numbers (*n*) are included in the respective figure legends.

## Supplementary information

Supplementary Table S1

Supplementary Table S2

Supplementary Table S3

Supplementary Data

## Data Availability

Mass spectrometry data have been deposited in ProteomeXchange (http://www.proteomexchange.org) with the accession code PXD028863. All data generated or analyzed during this study are included in this published article and its Supplementary files and available from the corresponding authors on request.
