# Supplementary material for: PURPL represses autophagic cell death to promote cutaneous melanoma by modulating ULK1 phosphorylation
Source: Cell Death Dis. 2021 Nov 10;12(11):1070. doi: 10.1038/s41419-021-04362-8 (PMC8581000; doi:10.1038/s41419-021-04362-8)
Supplement: Supplementary file 4 — Supplementary Data [file 41419_2021_4362_MOESM4_ESM.pdf]

Supplementary Figure S1

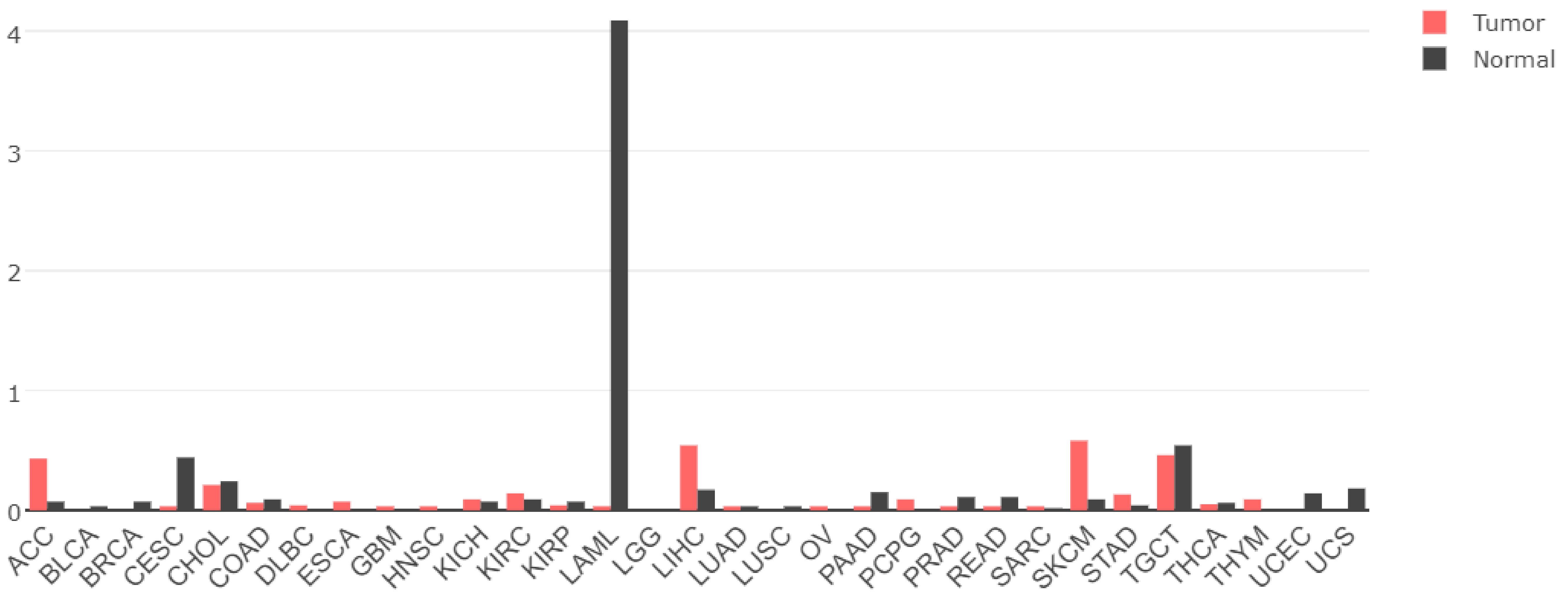

Supplementary Figure S1. Normalized PURPL expression levels in different types of cancer. The diagram showing the expression levels of PURPL in different types of cancer were analyzed and generated in Gene Expression Profiling Interactive Analysis (http://gepia.cancer-pku.cn/) using TCGA as data source.

Supplementary Figure S2

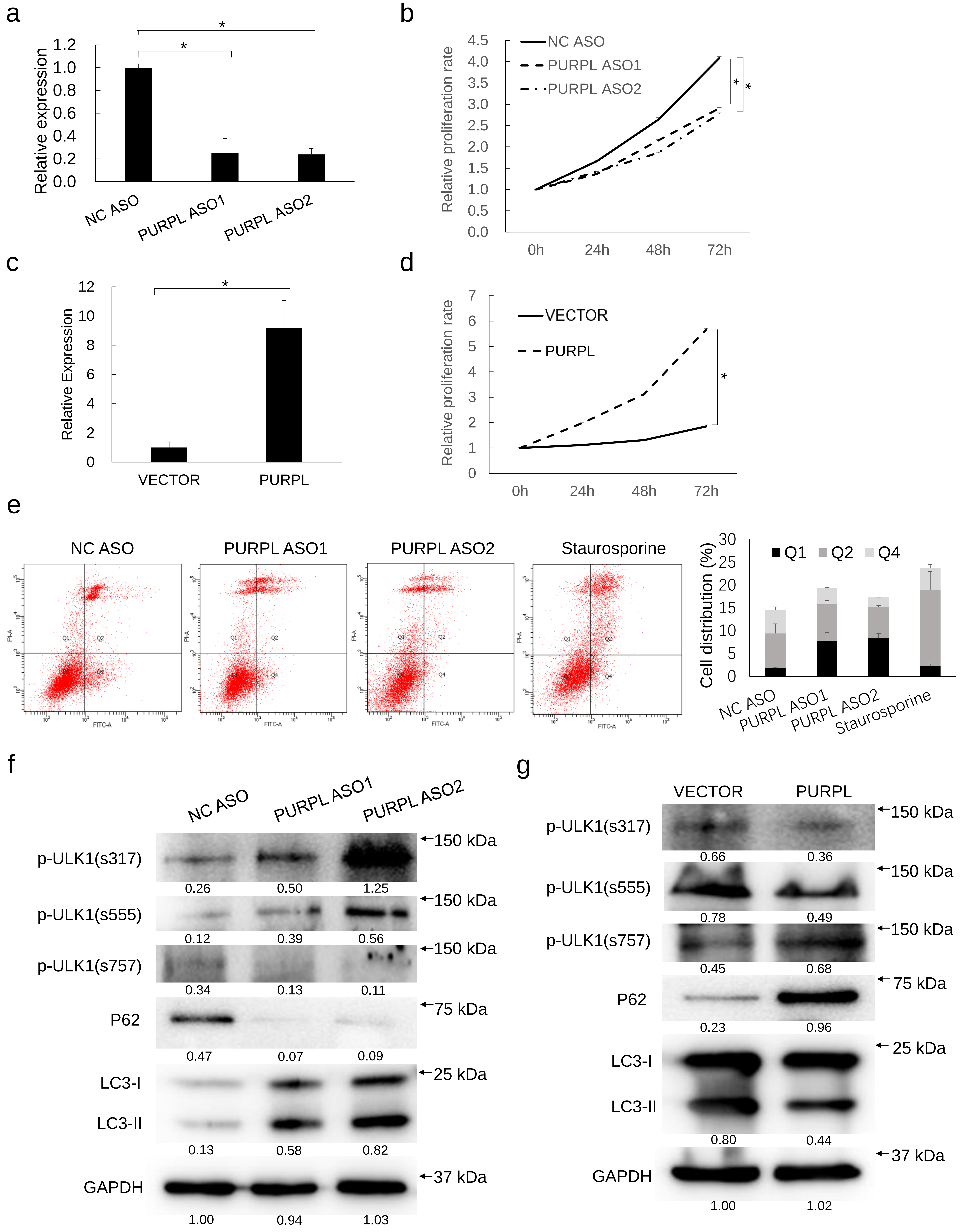

Supplementary Figure S2. PURPL promotes cell proliferation, migration and invasiveness in melanoma cells. (a) PURPL RNA expression was detected by qPCR after depletion of PURPL by ASOs in SK-MEL-28 cells. (b) Measurement of cell proliferation by CCK-8 assay was performed in SK-MEL-28 cells treated with ASOs targeting PURPL. (c) PURPL RNA expression was detected by qPCR after overexpression of PURPL in SK-MEL-28 cells. (d) Measurement of cell proliferation by CCK-8 assay was performed in SK-MEL-28 cells overexpressing PURPL. (e) The cell distribution was detected by flow cytometry with Annexin V/PI double staining in SK-MEL-28 melanoma cells after depletion of PURPL. Cells treated with 200nM Staurosporine for 24h were used as apoptosis control. (f,g) LC3B, P62 and ULK1 phosphorylations was detected in response to PURPL knockdown or overexpression by Western blot in SK-MEL-28 cells. GAPDH were also detected as control. One-Way ANOVA and Dunnett's multiple comparison test. Means ± s.d. *$p < 0.05$, **$p < 0.01$, ***$p < 0.001$.

Supplementary Figure S3

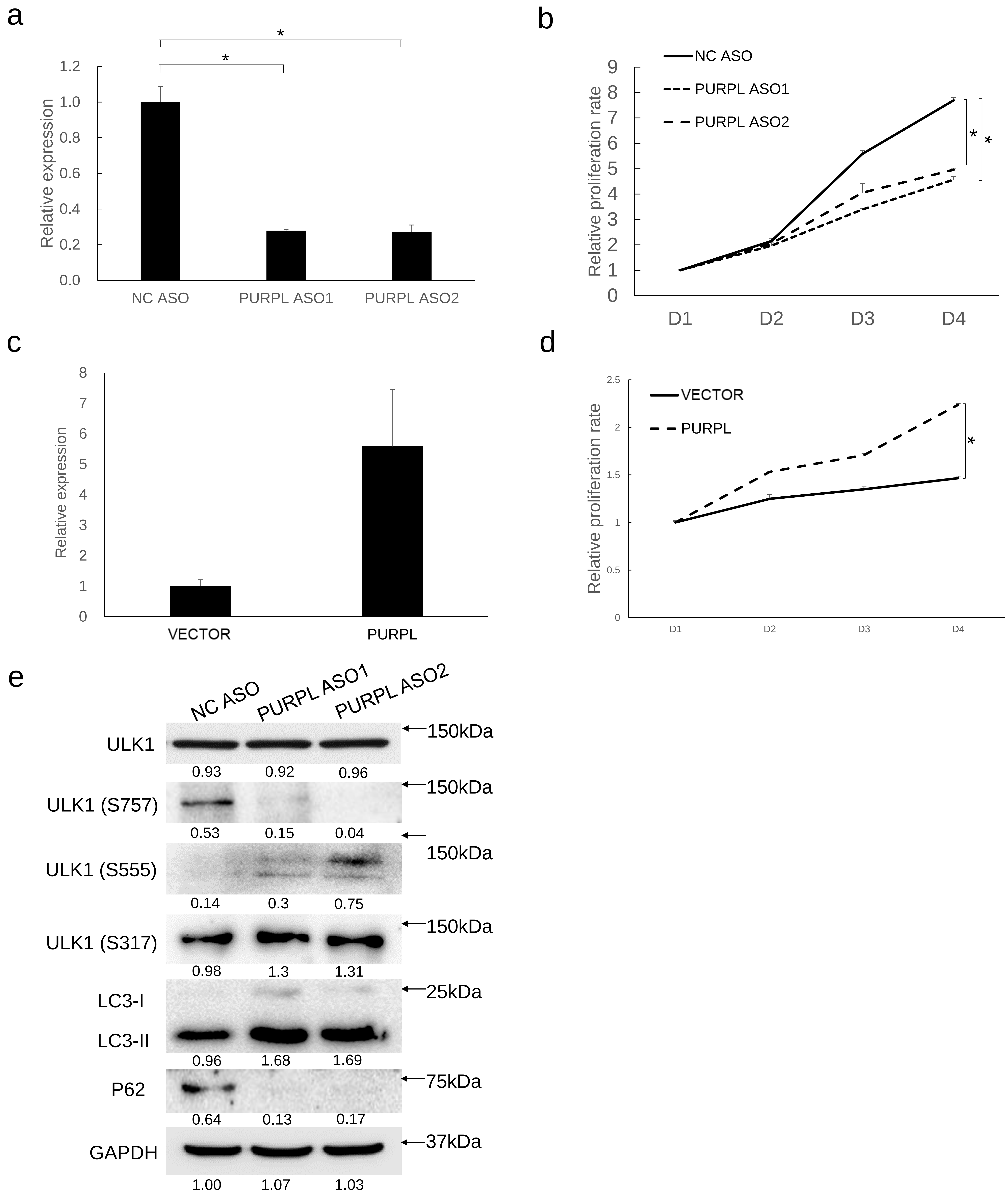

Supplementary Figure S3. PURPL functions as oncogene to promote cell proliferation and repress autophagy in SK-MEL-1 melanoma cells. (a,c) PURPL RNA expression was verified after depletion of PURPL using ASOs or overexpression of PURPL in SK-MEL-1 cells. (b,d) Measurements of cell proliferation by CCK-8 assay was performed in SK-MEL-1 cells treated with ASOs targeting PURPL or PURPL overexpression. Each experiment was performed at least in triplicate and data are presented as mean ± s.d. One-Way ANOVA and Dunnett's multiple comparison test were used to analyze the data (*$p < 0.05$, **$p < 0.01$, ***$p < 0.001$). (e) Different phosphorylations forms of ULK1, LC3B and P62 were detected by Western blot. ULK1 and GAPDH were also detected as controls.

Supplementary Figure S4

a

ULK1 peptide : LTDLLLK. t = 31.61 min

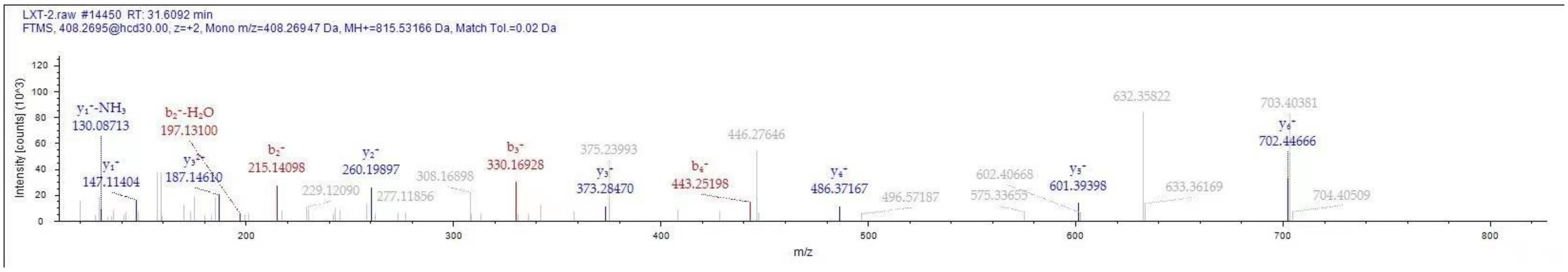

b

mTOR peptide : VLDIIR. t = 28.33 min

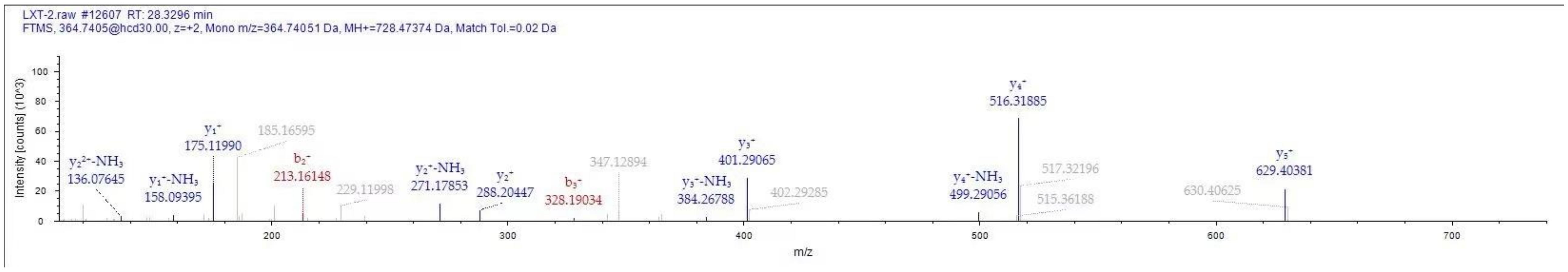

Supplementary Figure S4. Peptides of ULK1 and mTOR identified by HPLC-MS. (a, b) The amino acid sequences of ULK1 and mTOR peptides were identified by high performance liquid chromatography-mass spectrometry (HPLC-MS) analysis of the protein mix precipitated by *in vitro*-transcribed Biotin-labelled PURPL RNA.

Supplementary Figure S5

A375 cell line authentication

Figure 1. STR profiles of A-375 cell line

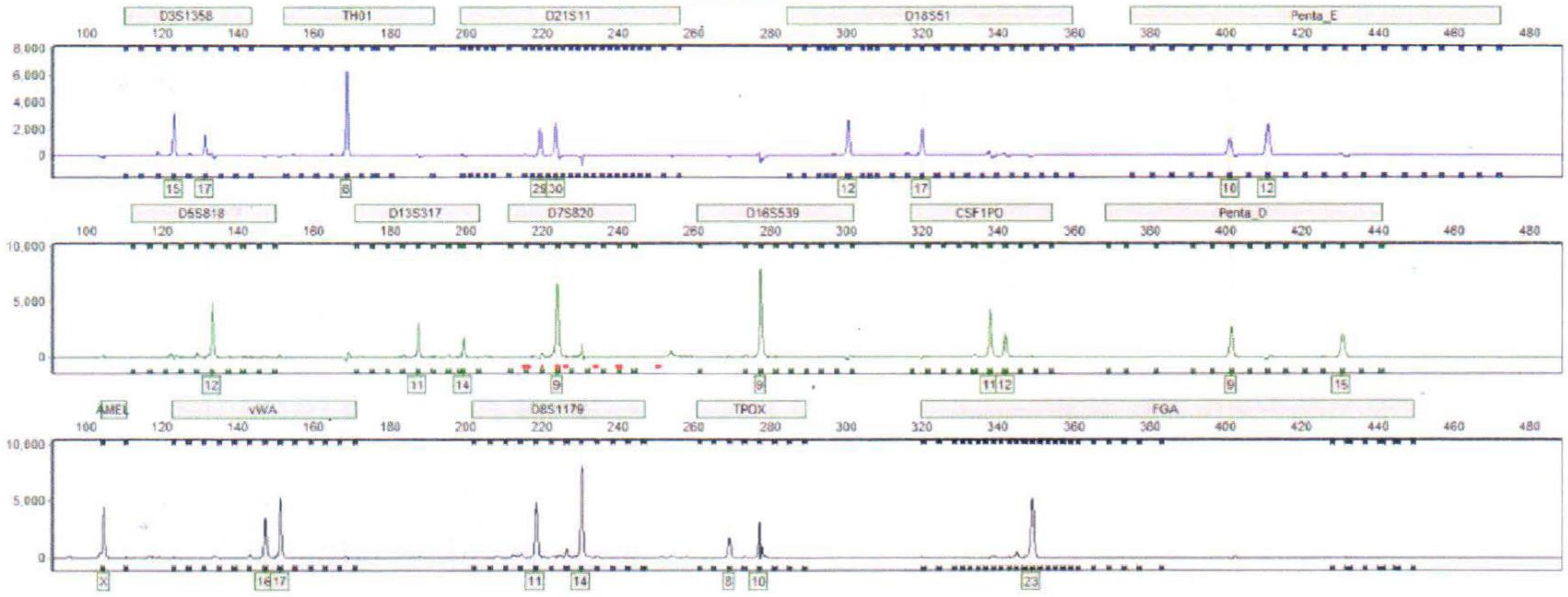

Table 1. STR profiles of A-375 cell line

	Allele1	Allele2
D3S1358	15	17
TH01	8	
D21S11	29	30
D18S51	12	17
Penta_E	10	12
D5S818	12	
D13S317	11	14
D7S820	9	
D16S539	9	
CSF1PO	11	12
Penta_D	9	15
AMEL	x	
vWA	16	17
D8S1179	11	14
TPOX	8	10
FGA	23	

Figure 2. Search result in ATCC database

SEARCH THE STR DATABASE

As part of our continuing efforts to characterize and authenticate the cell lines in the Cell Biology collection, ATCC has developed a comprehensive database of short tandem repeat (STR) DNA profiles for all of our human cell lines. View our brief tutorial before starting.

1. STR Profiling Analysis
2. Matching Algorithm
3. Interrogating the Database

Showing 1-- 5 Of 5

PageSize: 100 ▾

Add to Cart	%Match	ATCC® Number	Designation	D5S818	D13S317	D7S820	D16S539	vWA	TH01	AMEL	TPOX	CSF1PO
☐	100.0	CRL-1872	A375.S2MelanomaHuman	12	11,14	9	9	16,17	8	X	8,10	11,12
☐	100.0	CRL-1619	A375MelanomaHuman	12	11,14	9	9	16,17	8	X	8,10	11,12
☐	100.0	CRL-3222	A375-MA1MelanomaHuman	12	11,14	9	9	16,17	8	X	8,10	11,12
☐	100.0	CRL-3223	A375-MA2MelanomaHuman	12	11,14	9	9	16,17	8	X	8,10	11,12
☐	100.0	CRL-3224	A375PMelanomaHuman	12	11,14	9	9	16,17	8	X	8,10	11,12

Add to Cart   Export to Excel

Supplementary Figure S6

SK-MEL-28 cell line authentication

检测项目： STR

送检单位： 信裕生物

检测方法： 用 Axygen 的基因组抽提试剂盒提取 DNA，采用 20- STR 扩增方案扩增，在 ABI 3730XL 型遗传分析仪上对 STR 位点和性别基因 Amelogenin 进行检测。

检 测 结 果

(一) 检验基本情况

	多等位基因	匹配细胞系	细胞库	EV 值	匹配说明
20170821-03	有	SK-MEL-28	ATCC	0.81	基本匹配

样本基因型检验结果

- 多等位基因指三等位及以上基因现象。
- 本次检测各细胞分型结果良好。

(二) 各样本描述

- **20170821-01**：该株细胞 DNA 分型在细胞系检索中找到**基本匹配**的细胞系，ATCC 数据库显示细胞名为 **SK-MEL-28**，细胞号对应 **HTB-72**。本次检测在该细胞系中**发现多等位基因**。

备注： 待测细胞系与收录于 ATCC, DSMZ, JCRB 和 RIKEN 数据库的细胞系 STR 数据进行比对，未收录于以上细胞库的细胞系将无法匹配。

(三) 样本分型结果

细胞 20170821-01 的 STR 位点和 Amelogenin 位点的基因分型结果

Loci	送检细胞 STR 信息			细胞库细胞 STR 信息		
	送检细胞名：sk-mel-28			细胞库细胞名：sk-mel-28		
	Allele1	Allele2	Allele3	Allele1	Allele2	Allele3
D5S818	13	13		11	13	
D13S317	11	12		11	12	
D7S820	10	11		10	10	
D16S539	9	12		9	12	
VWA	16	19		16	19	
TH01	7	7		7	7	
AMEL	X	Y		X	Y	
TPOX	8	12		8	12	
CSF1PO	10	10		10	12	
FGA	19	19				
D21S11	28	29				
D18S51	12	16				
D8S1179	13	13				
D3S1358	16	18				
PENTAE	8	12				

其 他 说 明

(一) 分型方案及位点分布

	方案 1	方案 2	方案 3	方案 4
1	TH01	TPOX	D3S1358	AMEL
2	D12S391	VWA	D13S317	D5S818
3	D7S820	D8S1179	D6S1043	D2S1338
4	CSF1PO	PENTAD	D16S539	D21S11
5	FGA		D19S433	D18S51
6	PENTAE			

实验方案及位点

(二) STR 数据库比对

本公司采用 DSMZ tools 进行细胞系比对，其中包含来自于 ATCC, DSMZ, JCRB 和 RIKEN 数据库的 2455 个细胞系 STR 数据。如果待检测细胞未收录于以上细胞库或这是自行建立的新细胞系将无法进行比对，用户需根据细胞分型结果自行与其他数据库进行比对。

签发日期： 2017 年 08 月 29 日

上海信裕生物科技有限公司

Figure 1. STR profiles of SK-MEL-28 cell line

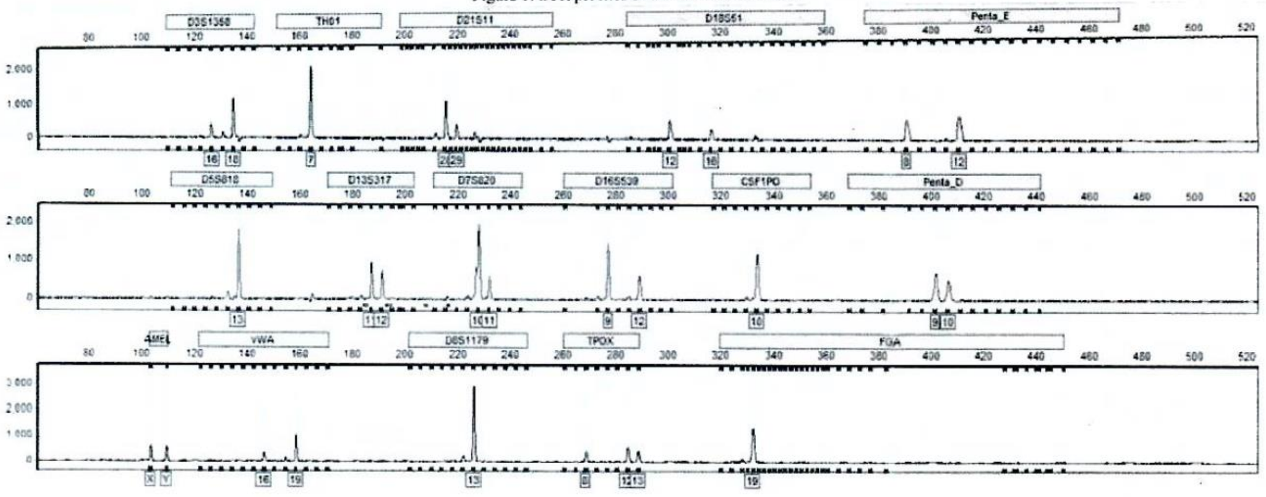

信裕细胞库支原体检测报告

细胞名称：SK-MEL-28

检测日期：2021/8/22

检测原理：

《发光法支原体检测试剂盒》通过检测支原体含有的特异性酶的活性以达到检 测体外培养的哺乳动物细胞是否被支原体污染的目的。在支原体裂解后，该支原体特异性的 酶，在底物存在下，具有将 ADP 转化成 ATP 的功能。由于荧光素酶(Luciferase)催化底 物荧光素(Luciferin)产生光的反应需要 ATP 的参与，支原体特异性酶催化产生的 ATP 含 量，可以通过该反应转化成生物发光(Bioluminescence)信号，该信号可以使用专门的发光 检测仪(Luminometer)或具有发光检测功能的多功能酶标仪进行检测，发光的强度与 ATP 的含量成正比。

具体的反应如下：

通过比较细胞培养上清和未用于细胞培养而成分完全相同的培养液二者的支原体特异性酶的含量，即可知道培养的细胞是否被支原体污染。

实验基本步骤：

1、吸取 50μL 阴性对照或者已经经过低速离心去除细胞和经过高速离心替换成阴性对照的培养液的待测样品，放入白色或者黑色不透明的 96 孔板内，加入 50μL 试剂 A，室温反应 15 分钟。

2、加入 50μL 试剂 B，室温反应 3 分钟后，在多功能酶标仪或者发光检测仪(Luminometer)上，以仪器默认的参数进行发光值的检测，5 分钟内，连续测 5 次阴性对照和待测样品的发光值，计算各自的平均值。

结果判断：

计算待测样品的发光平均值与阴性对照的发光平均值的比值：

1、如果比值>1.2，说明待测样品有支原体污染（阳性）。

2、如果比值在 1.1-1.2 之间，说明待测样品可疑有支原体污染（可疑阳性）。但该样品需要继续培养 24-48 小时后，重新检测。如果继续培养 24-48 小时后，重新检测的比值仍然在 1.1-1.2 之间，应该判为阴性。

3、 如果比值<1.1，说明待测样品无支原体污染（阴性）。

检测次数	1	2	3	4	5
检测数据	384	418	369	402	383
阴性对照	388	423	409	434	387

结论： 支原体检测为阴性[√] 阳性[ ]。

上海信裕生物科技有限公司
电话：021-60526354
网址：https://www.shxybio.net/

Supplementary Figure S7

SK-MEL-1 cell line authentication

附表 1：细胞株 SK-MEL-1 的 STR 位点和 Amelogenin 位点的基因分型结果

细胞 SK-MEL-1（图片编号为 PC228）		
Marker	***Allele 1***	***Allele 2***
D3S1358	14	16
**THO1**	6	6
D21S11	29	32.2
D18S51	13	16
Penta E	7	21
**D5S818**	12	13
**D13S317**	11	11
**D7S820**	12	12
**D16S539**	11	12
**CSF1PO**	12	13
Penta D	11	13
**AMEL**	X	Y
**vWA**	16	17
D8S1179	13	16
**TPOX**	11	11
FGA	18	20
D6S1043	11	11
D2S1338	19	23
D12S391	18	20
D19S433	15	16.2
D1S1656	12	17.3

SK-MEL-1 细胞出库质检单

一、产品信息

1. 细胞名称：SK-MEL-1（人皮肤黑色素瘤细胞）
2. 细胞货号：CL-0440
3. 出库日期：2019-12-27

二、检测项目及结果

检测项目	检测结果	
生长特性（贴壁/悬浮）	悬浮	
细胞形态	球形	
细胞密度	＞75%	
细胞纯度	——	
细胞总量	~1×10⁶/Cells	
细胞活力	＞95%	
HIV-1	有☐	无☑
HBV	有☐	无☑
HCV	有☐	无☑
支原体	有☐	无☑
细菌	有☐	无☑
酵母	有☐	无☑
真菌	有☐	无☑

三、质检员及质检日期

质检员：王帅　　质检日期：2019-12-24

质检专用章　　质量合格，准予放行

网站：www.procell.com.cn
电话：400-650-3656
邮箱：sales@procell.com.cn

附图 2：SK-MEL-1 细胞 STR 位点和 Amelogenin 位点的基因分型结果

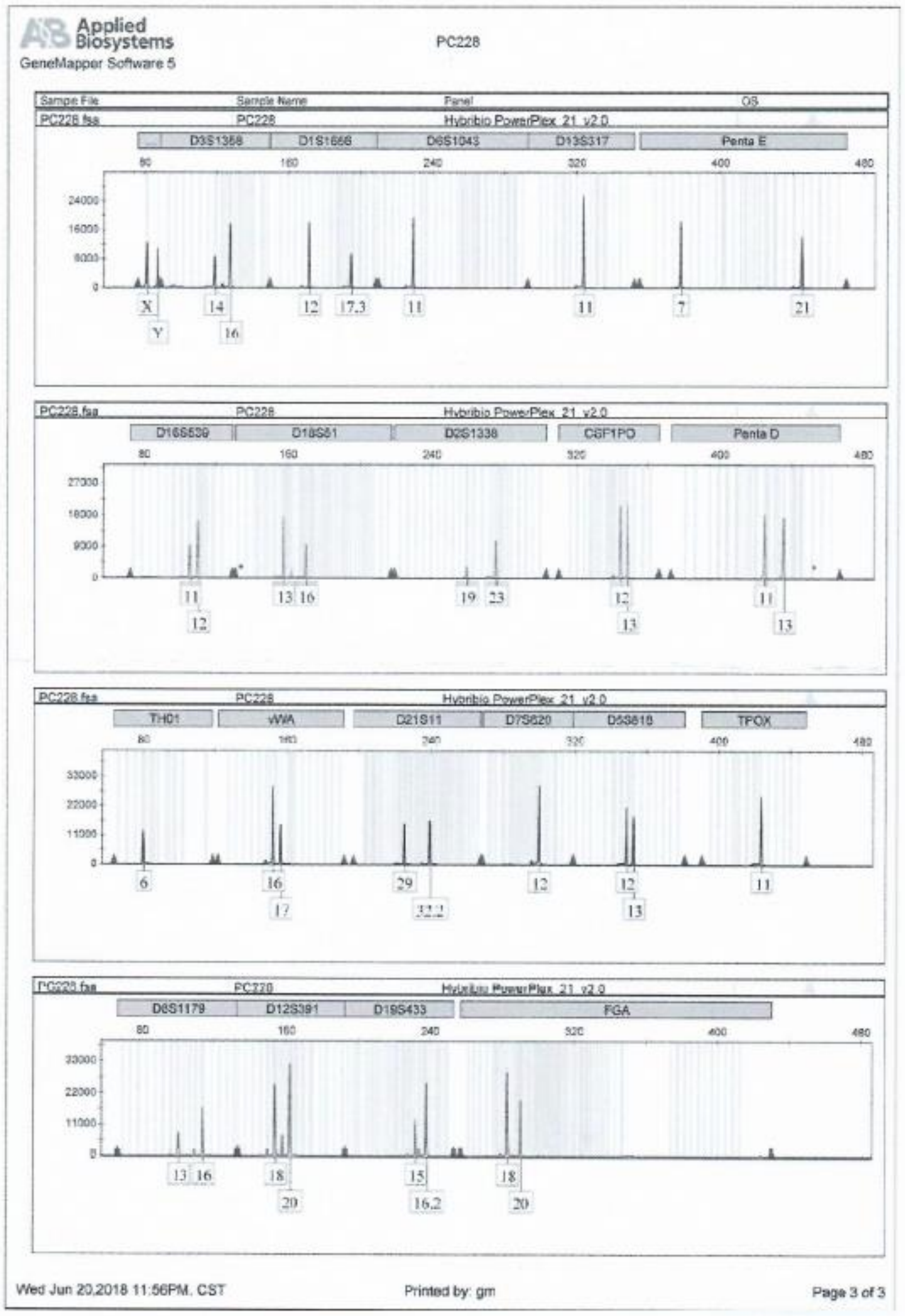

附图 1：ATCC 官网 SK-MEL-1 细胞 STR 位点信息

SK-MEL-1 (ATCC® HTB-67™)

Organism: Homo sapiens, human / Cell Type: Melanoma / Tissue: malignant mel... / Disease: malignant melanoma

GENERAL INFORMATION | CHARACTERISTICS | CULTURE METHOD | SPECIFICATIONS

STR Profile

Amelogenin: X,Y
CSF1PO: 12,13
D13S317: 11
D16S539: 11,12
D5S818: 12,13
D7S820: 12
THO1: 6
TPOX: 11
vWA: 16,17
